# Statistical Analysis and Kinematic Assessment of Upper Limb Reaching Task in Parkinson’s Disease

**DOI:** 10.3390/s22051708

**Published:** 2022-02-22

**Authors:** Alfonso Maria Ponsiglione, Carlo Ricciardi, Francesco Amato, Mario Cesarelli, Giuseppe Cesarelli, Giovanni D’Addio

**Affiliations:** 1 Department of Electrical Engineering and Information Technology, University of Naples Federico II, 80125 Naples, Italy; alfonsomaria.ponsiglione@unina.it (A.M.P.); carloricciardi.93@gmail.com (C.R.); framato@unina.it (F.A.); cesarell@unina.it (M.C.); 2 Institute of Care and Scientific Research of Telese of ICS Maugeri SPA SB, 82037 Telese Terme, Italy; gianni.daddio@icsmaugeri.it; 3 Department of Chemical, Materials and Production Engineering, University of Naples Federico II, 80125 Naples, Italy

**Keywords:** motion analysis, reaching movements, biomedical signal processing, kinematic features, Parkinson’s disease

## Abstract

The impact of neurodegenerative disorders is twofold; they affect both quality of life and healthcare expenditure. In the case of Parkinson’s disease, several strategies have been attempted to support the pharmacological treatment with rehabilitation protocols aimed at restoring motor function. In this scenario, the study of upper limb control mechanisms is particularly relevant due to the complexity of the joints involved in the movement of the arm. For these reasons, it is difficult to define proper indicators of the rehabilitation outcome. In this work, we propose a methodology to analyze and extract an ensemble of kinematic parameters from signals acquired during a complex upper limb reaching task. The methodology is tested in both healthy subjects and Parkinson’s disease patients (N = 12), and a statistical analysis is carried out to establish the value of the extracted kinematic features in distinguishing between the two groups under study. The parameters with the greatest number of significances across the submovements are duration, mean velocity, maximum velocity, maximum acceleration, and smoothness. Results allowed the identification of a subset of significant kinematic parameters that could serve as a proof-of-concept for a future definition of potential indicators of the rehabilitation outcome in Parkinson’s disease.

## 1. Introduction

Neurological disorders are known to have an extremely negative impact on a person’s quality of life and to be among the leading cause of disability and death globally [1,2,3]. In particular, those labelled as neurodegenerative diseases, i.e., characterized by progressive neuro losses, are seeing an increase in their prevalence and incidence in the global population [1,4]. Alzheimer’s disease, Parkinson’s disease, multiple sclerosis, as well as other cognitive impairments, have a considerable impact on public health expenditure and, despite the efforts to establish new and effective pharmacological therapies, the current drug-based treatments still present limited advantages [2,3,4,5]. Therefore, physical exercise approaches have been proposed as promising strategies to support the treatment of such neurodegenerative disorders [2,3,4,5].

The benefit of motor rehabilitation tasks proved to be particularly helpful in Parkinson’s disease, since the physical training was demonstrated to be effective in improving both motor and non-motor Parkinson’s symptoms [6]. Some studies specifically focused on discovering novel rehabilitation tasks to improve the upper limb function in Parkinson’s disease patients [6,7,8,9,10,11]. However, in the case of upper limbs, it is worth highlighting that they are composed of many joints, which are the most flexible in the human body, thus bringing a high degree of freedom of movement. The intrinsic complexity of the joints’ control mechanisms makes the assessment of the therapeutic impact of the physical exercise a very challenging task. Some studies use grasping tasks to understand the kinematic and dynamic aspects of the spontaneous movement of the human arm. Some of these studies have identified common kinematic features and patterns that characterize spontaneous human movements [12,13,14]. In particular, it has been observed that when people move their arms from one point in space to another, they tend to create a straight, regular path without interrupting acceleration. The model, first proposed by Hogan in 1984 [15,16], describes the movements of a healthy person from a theoretical point of view and is often used by physicians to describe spontaneous arm movements.

Several biomechanical parameters have been proposed in the scientific literature to date for assessing the quality of movement in healthy and diseased subjects in different rehabilitation settings and tasks [16,17,18,19,20,21,22,23,24,25,26,27,28,29,30,31]. Moreover, several studies have aimed to exploit motion analysis data and compare different instrumentations for diagnostic purposes [32,33]. However, there is still the lack of objective and effective quantitative kinematic and dynamic indicators of rehabilitation outcome as well as the lack of standardized rehabilitation protocols to restore motor function in Parkinson’s disease.

The objective of this study is to propose a methodology to estimate kinematic features from upper limb reaching tasks that, taken as a whole, could serve as a basis to estimate a quantitative rehabilitation outcome. The methodology is tested in both healthy subjects and Parkinson’s disease affected patients to provide a proof-of-concept of the adopted strategy, and the most significant kinematic parameters distinguishing the two groups of subjects are identified and discussed.

## 2. Materials and Methods

Retrospective data and signals on 12 subjects, of which six were healthy subjects (from a population of healthy individuals with: age = 40.0 ± 5.7 years old; BMI = 26.0 ± 4.0), and six were Parkinson patients (from a population of pathological individuals with: age = 51.7 ± 13 years old; BMI = 27.6 ± 4.6), collected at the Institute of Care and Scientific Research of Telese Terme of ICS Maugeri SPA SB (Telese Terme, Italy), have been processed and analyzed in order to estimate and compare a set of kinematic parameters between the two groups. Each subject performed the task twice for a total of 24 signals.

**Figure 1:**
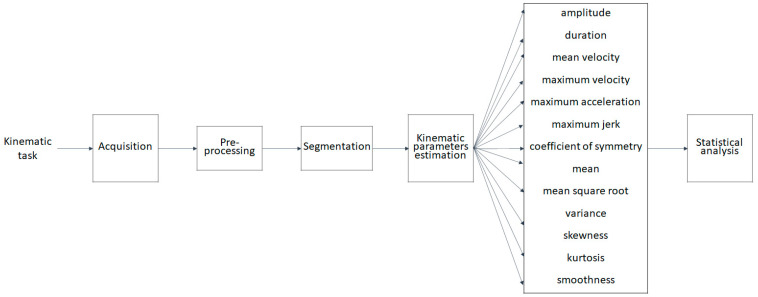
Methodological workflow.

Figure 1 displays the methodological workflow in brief, while each phase of the workflow is described in detail in the following paragraphs.

### 2.1. Kinematic Task and Acquisition

Angular displacement signals, acquired through goniometer sensors were acquired by implementing a kinematic task protocol consisting of four movements performed by the upper limb, as described earlier [34,35]. With respect to movement and for its correct interpretation, the human body can be divided into three anatomical levels: (I) Sagittal plane, the symmetric plane of the body; (II) frontal plane, perpendicular to the sagittal plane passing through the center of gravity (mass) of the body; (III) a horizontal plane, orthogonal to the other two and passing through the center of gravity of the body. In the proposed protocol, the movements are carried out along two axes, defining a horizontal reaching task and a vertical reaching task, in a two-dimensional plane starting from a reference position (from now on called “middle position” for the sake of simplicity). During the acquisition, the subject is in an upright position, with a straight trunk and neck and the gaze fixed on the central point of the plane on which the reaching movements are implemented. The kinematic protocol consists in the exploration of 4 positions: top, bottom, right, and left but can also be broken down into 8 distinct kinematic phases, four of elevation and lowering in the sagittal plane and four of extension and flexion in the horizontal plane, as listed in Table 1.

The goniometric sensors allowed the acquisition of spatial coordinates at a sampling rate of 20 Hz. The signals were then preprocessed as described in the following paragraph.

### 2.2. Preprocessing

Signals were preprocessed and processed by means of a custom-made program developed in Matlab (MathWorks, R2021a, Natick, MA, USA). The acquired signal was loaded and resampled, interpolated, filtered, and derived in order to obtain a clean manipulable signal, with all the kinematic descriptions: position, velocity (time derivative of the position, as from Equation (1)), acceleration (second time derivative of the position, as from Equation (2)), and jerk (third time derivative of the position, as from Equation (3)).

The latter was defined as the rate of the change of acceleration with respect to time, as described in our previous work [24]:

(1) $$v\left(t\right)=\int_{0}^{d}\left|\frac{dx}{dt}\right|$$

(2) $$a\left(t\right)=\int_{0}^{d}\left|\frac{d^{2}x}{dt^{2}}\right|$$

(3) $$J\left(t\right)=\int_{0}^{d}\left|\frac{d^{3}x}{dt^{3}}\right|$$

where *x*(*t*) is the position signal, d is the angular distance (expressed in degrees as measured through goniometric sensors) travelled by the arm during the exercise, and *v*(*t*), *a*(*t*), and *J*(*t*) are the velocity, acceleration, and jerk, respectively.

Before calculating *v*(*t*), *a*(*t*), and *J*(*t*), a resampling of the position signal *x*(*t*) occurs at 1 kHz, by means of spline interpolation. This step was implemented in view of the future developments of this study, which will aim at coregistering electromyography signals sampled at 1 kHz together with the signals acquired by means of motion sensors, as also further explained in the discussion section of this manuscript. Kinematic signals were smoothed with a zero-phase, fifth-order, low-pass Butterworth filter with an experimentally selected cutoff frequency of 1.5 Hz (Equation (5) shows the general frequency response of a fifth order Butterworth filter).

(4) $$\left|H\left(j\omega \right)\right|=\frac{1}{\sqrt{1+\omega^{2n}}}$$

where *n* is the order of the filter, and ω is the ratio between the signal frequency and the cutoff frequency. The proposed smoothing filter was chosen, since it has been largely applied for the preprocessing of kinematic signals, as also outlined in other works [36,37,38]. The derivations are carried out through the forward Euler method [39].

**Figure 2:**
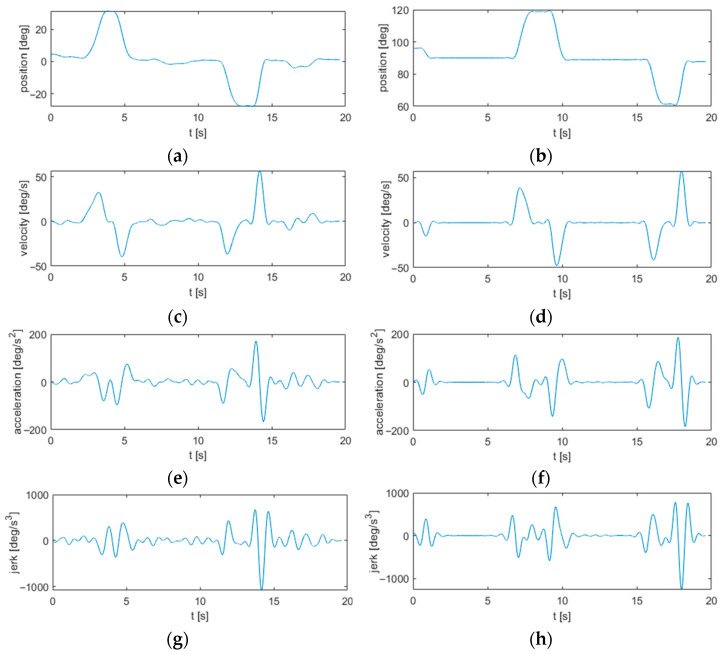
Preprocessing of motion signals from the horizontal reaching task: (**a**) position; (**c**) velocity; (**e**) acceleration; and (**g**) jerk. Preprocessing of motion signals from the vertical reaching task: (**b**) position; (**d**) velocity; (**f**) acceleration; and (**h**) jerk.

Figure 2 shows the output signals from the preprocessing phase.

The signals are used for the successive segmentation phase, as detailed in the following paragraph.

### 2.3. Segmentation

Velocity profiles were used to perform the segmentation for the extraction of sub-movements and the following kinematic analysis for each submovement for each patient.

After a filtering operation (for the removal of spurious peaks within the signal), the segmentation returned the time instants and the corresponding start and end positions of the movement. To discriminate the eight phases mentioned above, a movement detection algorithm was developed that performed a real control on the two velocity curves (obtained by deriving both the position signals) recognizing a movement where there are significant variations of the curve. In particular, the algorithm used a detection threshold, equal to 30% of the peak value of the absolute velocity curve. The value of the threshold was experimentally set at 30% of the maximum value of the absolute velocity (Equation (5)):

(5) $$Threshold=0.3*\max\left(\left|v\right|\right)$$

where *v* is the velocity signal (°/s).

The algorithm iteratively recorded the submovement onset and offset by calculating local maxima for those signal portions overcoming the threshold and local minima for those signal portions below the threshold, also inspired by the kinematic segmentation proposed in a previous work [36]. Briefly, the instant corresponding to the local minima preceding the threshold were labelled as sub-movement onsets. Similarly, the algorithm recognized the submovement offsets as the ones corresponding to the local maxima of the velocity profile following the threshold point. The process was iterated on both velocity curves (for both horizontal and vertical reaching tasks), so that the algorithm returned the starting and ending points of each single kinematic phase, allowing the system to obtain eight distinct velocity curves corresponding to the eight submovements.

**Figure 3:**
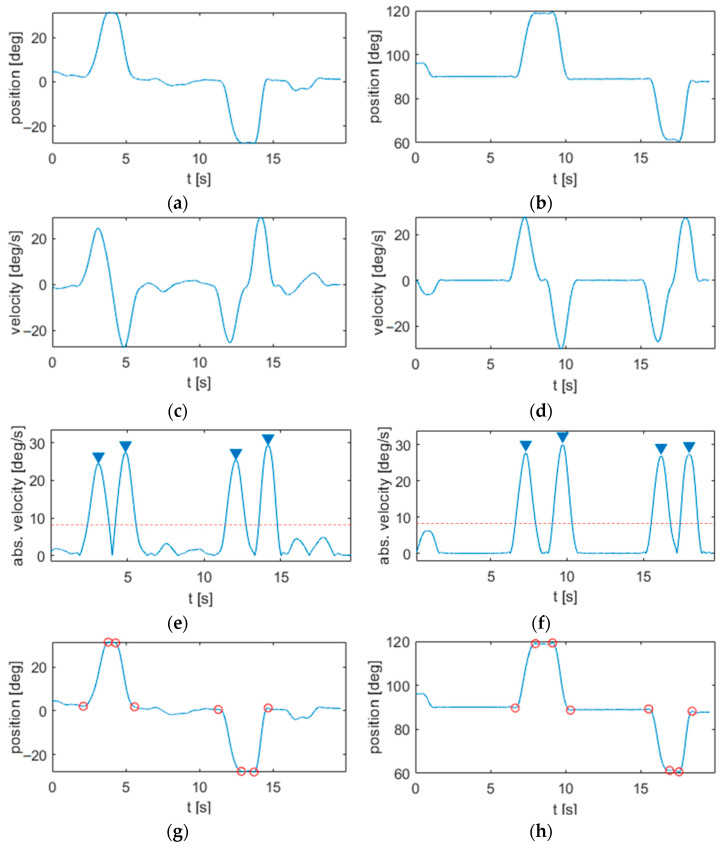
Segmentation of motion signals from the horizontal reaching task: (**a**) position; (**c**) velocity; (**e**) absolute velocity with maximum peaks (indicated with blue arrows) and threshold for peak detection (red dotted line); and (**g**) segmented position with indication of the onset and offset points of each submovement (red circles represent onsets and offsets of the submovements). Segmentation of motion signals from the vertical reaching task: (**b**) position; (**d**) velocity; (**f**) absolute velocity with maximum peaks (indicated with blue arrows) and threshold for peak detection (red dotted line); and (**h**) segmented position with indication of the onset and offset points of each submovement (red circles represent onsets and offsets of the submovements).

The segmentation steps are illustrated in Figure 3.

As shown in Figure 3, starting from the velocity and absolute velocity profiles, all the points above a previously defined threshold (indicated by the red dotted line in Figure 3e,f), where the absolute velocity reached its maximum values were identified and marked (blue triangles in Figure 3e,f). Then, the time instants when the velocity profile reached its local minima and maxima, respectively, right before and right after the defined threshold (equal to 30% of the maximum velocity peaks) were identified and marked as the onsets and offsets of each submovement (red circles in Figure 3g,h).

### 2.4. Kinematic Parameters Estimation

The following kinematic parameters were extracted for each of the eight submovements:

- amplitude: representing the rotation amplitude of the executed submovement, i.e., the angular distance (expressed in degrees as measured through goniometric sensors) travelled by the arm during the execution of a submovement;
- duration: obtained from the difference between the end and start point of the submovement (expressed in seconds);
- mean velocity (v_mean): obtained as the ratio between the amplitude and the duration of the submovement (expressed in degrees per second);
- maximum velocity (v_max): maximum value of the velocity within the submovement (expressed in degrees per second);
- maximum acceleration (a_max): maximum value of the acceleration within the submovement (expressed in degrees per squared second);
- maximum jerk (jerk_max): maximum value of the jerk within the submovement (expressed in degrees per cubic second);
- coefficient of symmetry (symmetry): obtained as the ratio between the duration of the deceleration phase and the duration of the acceleration phase within the sub-movement (expressed in dimensionless units), as described in our previous studies [18,25,40,41];
- mean value of the position (p_mean): calculated as the mean of the Gaussian-like morphology of the velocity profile of the sub-movement, as described in our previous studies [18,25,40,41];
- mean square root value of the position (p_root_mean): calculated as the mean square root value of the Gaussian-like morphology of the velocity profile of the submovement, as described in our previous studies [18,25,40,41];
- variance: calculated as the variance of the Gaussian-like morphology of the velocity profile of the submovement, as described in our previous studies [18,25,40,41];
- skewness of the velocity profile (skewness): calculated as the skewness of the Gaussian-like morphology of the velocity profile of the submovement, as described in our previous studies [18,25,40,41];
- kurtosis of the velocity profile (kurtosis): calculated as the kurtosis of the Gaussian-like morphology of the velocity profile of the submovement, as described in our previous studies [18,25,40,41];
- smoothness: calculated as the integral of the third time derivative of the position over the submovement, as described in our previous studies [18,25,40,41];

The obtained kinematic parameters were used to carry out a statistical analysis to compare the two groups of subjects considered in this study, as described in the following paragraph.

### 2.5. Statistical Analysis

Data management and statistical analyses were performed by means of Excel (MS Office) and IBM SPSS Statistics (v27). The Shapiro-Wilk test was carried out for the normality check of the distribution of data. It was used as a reference since it proved to be more appropriate method for small sample size dataset rather than Kolmogorov-Smirnov test [42]. The Student’s *t*-test and the U-Mann Whitney test (Confidence Interval: 95%, i.e., α = 0.05, two sided tests for unpaired data) were adopted, for normally and non-normally distributed data, respectively, to compare the central tendency of the data between the two groups of subjects (healthy subjects and Parkinson’s disease affected patients) for each estimated kinematic parameter. The Levene’s test was used to assess the homoscedasticity of the data before applying the *t* test.

The purpose of the statistical analysis was to identify those factors that were statistically significant in discriminating between the two groups of subjects and that could serve as helpful indicators of the rehabilitation outcome in Parkinson patients.

## 3. Results

### 3.1. Signal Processing

**Figure 4:**
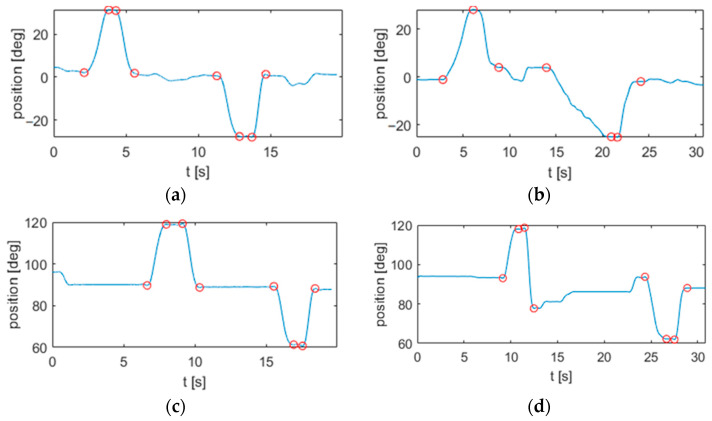
Segmentation of motion signals from the horizontal reaching task in a (**a**) healthy subject and (**b**) Parkinson patient. Segmentation of motion signals from the vertical reaching task in a (**c**) healthy subject and (**d**) Parkinson patient.

Figure 4 displays segmented signals from both a healthy and a pathological subject.

The segmentation procedure allows the recognition of the submovements even in the case of visible motion artifacts and tremor due to Parkinson’s disease (Figure 4b,d). This also allowed the proper estimation of the kinematic parameters for each submovement in both groups of patients, as described in the following paragraph.

### 3.2. Statistical Analysis and Classification

Table 2 shows the results from the statistical analysis for sub-movement 1 (additional analyses carried out for the other submovements are reported in Tables S1–S8 of the Supplementary Material).

Most of the parameters were statistically significant in discriminating between healthy and Parkinson subjects, except for jerk_max, p_mean, and p_root_mean, which were not statistically significant.

**Figure 5:**
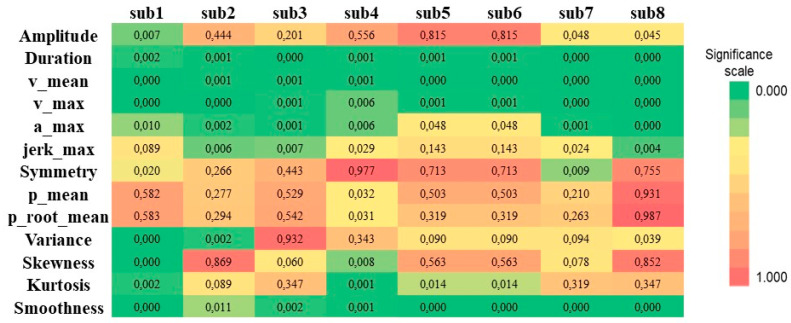
Heatmap showing the distribution of the *p*-values for each parameter and per each submovement. Each column represents a submovement while each row indicates the extracted kinematic parameter. Features with strong statistical significance are reported in green, while weak and strongly weak *p*-values are reported in yellow and red, respectively.

A heatmap was also designed to show the distribution of the *p*-value per each parameter and per each submovement (Figure 5).

A statistical analysis was carried out to compare the average values of the kinematic parameters calculated for all the submovements, according to the following equation:

(6) $$average\;kp=\frac{1}{8}\overset{8}{\underset{i=1}{\sum }}kp_{i}$$

where *average kp* is the average value of a generic kinematic parameter, and *kp_i_* is the value of the kinematic parameter calculated on the i-th submovement (related results are reported in in Table S9 of the Supplementary Material).

The statistical analysis on the average values of the kinematic parameters showed that the most significant indicators for distinguishing the two groups were the amplitude, the maximum velocity, the skewness, the kurtosis, and the smoothness. The maximum accelerations and the root mean square proved to be significant as well.

### 3.3. Kinematic Parameter Selection

**Figure 6:**
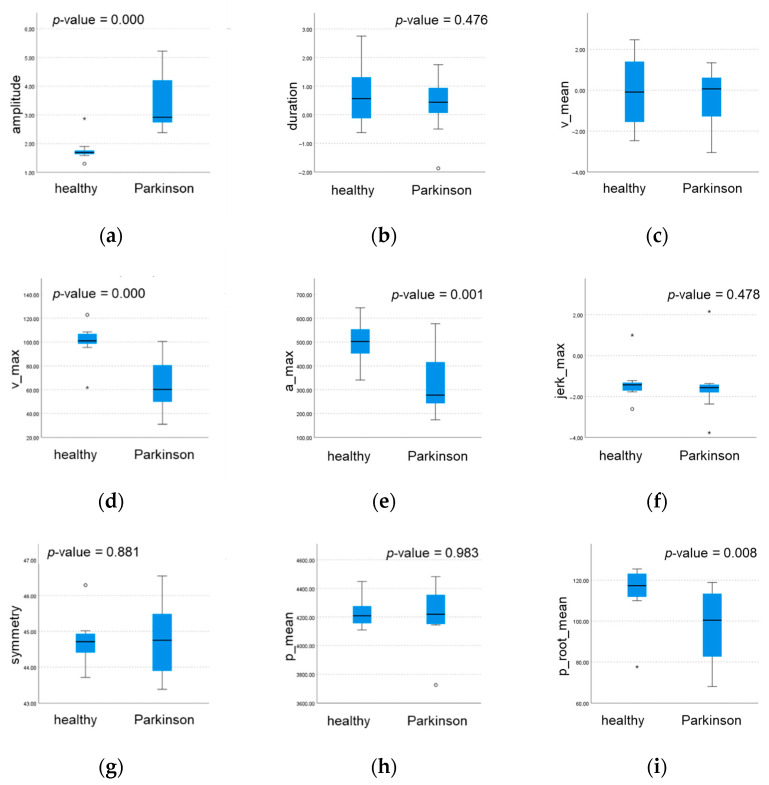

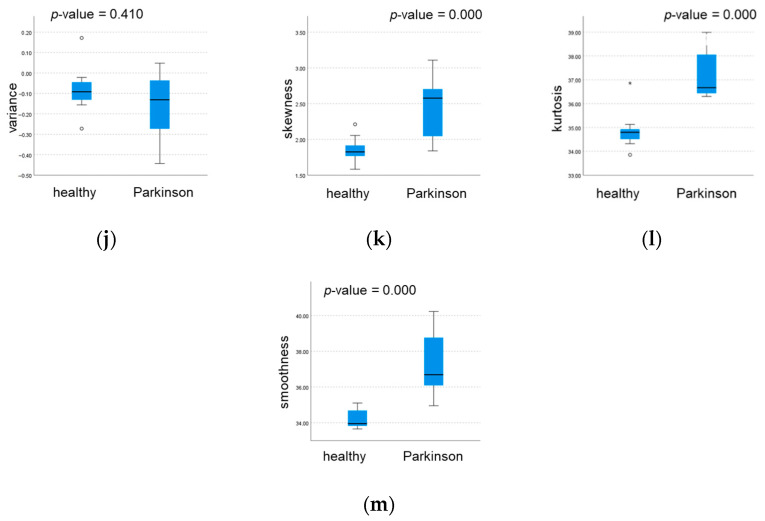
Boxplots for comparing the median values calculated on each averaged kinematic parameter for both the groups: (**a**) amplitude; (**b**) duration; (**c**) mean velocity; (**d**) maximum velocity; (**e**) maximum acceleration; (**f**) maximum jerk; (**g**) symmetry; (**h**) mean position; (**i**) mean square root of the position; (**j**) variance; (**k**) skewness; (**l**) kurtosis; and (**m**) smoothness. Circles (◦) and stars (*) represent outliers and extreme outliers (more than three times the interquartile range below the first quartile or above the third quartile) respectively.

The results regarding the averaged parameters can be seen in the boxplot displayed in Figure 6, which could support the selection of the most valuable kinematic parameters able to distinguish between the healthy and the diseased subjects.

When averaged on the submovements, the number of estimated kinematic parameters that were statistically significant was reduced with respect to the case when only a single submovement was taken into consideration (as shown in Table 2), thus suggesting that, while each submovement brings its own contribution (as detailed in Tables S1–S8 of the Supplementary Material), the average values of the kinematic parameters should be used as a more reliable indicator of the overall rehabilitation task outcome. In particular, those average kinematic parameters that were identified as most significant (i.e., amplitude, maximum velocity, skewness, kurtosis, and smoothness) in distinguishing the two groups of subjects could be taken into account to calculate a composite indicator of the rehabilitation outcome.

## 4. Discussion

This study has proposed a novel alternative methodology to estimate a quantitative rehabilitation outcome for upper limb reaching tasks. Specifically, after a signal processing routine based on custom-made software, thirteen kinematic parameters, related to upper limb tasks, were estimated and, subsequently, statistically analyzed. From these analyses, it has been found that a subset of features (namely, maximum velocity, skewness, kurtosis, and smoothness) effectively allowed, in the first instance as proof-of-concept, distinguishing the reaching movements performed by healthy and Parkinson’s Disease subjects, further confirming the preliminary evidence highlighted in a previous work [18].

The composition of purpose, experimental setup (signal acquisition and processing workflows), and the promising findings obtained represent, to the best of the authors’ knowledge, a yet unexplored strategy in this field, which set this study apart from others. Although we cannot present both current and past studies, which can directly support (or conflict with) our findings, we can briefly discuss in greater detail several aspects of other scientific contributions on these topics.

To the best of our knowledge, Hasman et al. [43] reported on a methodology aimed at distinguishing Parkinson subjects from healthy subjects, which could be compared to the approach proposed in the present study. In particular, the authors’ aim was to investigate subjects’ posture/stability by means of a functional reach test during which an accelerometer (previously positioned at the patients’ lower back) was used to acquire raw signals. The outcomes of regression analyses demonstrated kinematic parameters such as functional reach distance, anterior–posterior acceleration, and mediolateral acceleration were statistically significant between the two groups. Nevertheless, since, perhaps, the protocol used by Hasman et al. did not allow consideration of submovement, a possible potential inference we can draw in the first instance is that our and Hassman et al. study agree that (overall) the acceleration of the reaching task may effectively represent a parameter to distinguish Parkinson’s Disease patients from healthy controls.

Another method presented in the literature to potentially find a quantitative rehabilitation outcome is the use of drawing tests. Toward this aim, Bai recently presented a pilot study showing the possibility of acquiring, using a custom-made inertial sensor, the process time-frequency spectra of a healthy subject [11]. Albeit the author did not assess the method performing a pilot comparison with unhealthy subjects, the conclusions in his work, namely, the strategy “could potentially work as a useful method and provide additional insights in clinical rehabilitation” [11], are in line with the outcomes of Nadeau et al. [6] who, in the context of a 12-week aerobic exercise training, conducted a similar analysis. In particular, the authors used a kinematic model to extract several kinematic parameters (related to antagonist response and activation during the upper limb movement) even from a target-directed fast simple reaction time task carried out on both healthy and Parkinson’s disease patients; the outcomes indicated the 12-week training helped improve the upper limb motor function of Parkinson’s disease patients [6].

In the same period, Ferraris et al. [10] developed a novel system, based mainly on a low cost RGB-Depth camera and on gloves with imprinted color markers, for the automated Assessment of Unified Parkinson’s Disease Rating Scale (UPDRS) upper limb tasks in Parkinson’s Disease. The authors effectively demonstrated that features (such as the maximum speed of supination/pronation step task) were significantly correlated to the UPDRS severity and the machine learning strategies investigated were effectively able to distinguish between healthy and pathological subjects with a very high accuracy, which decreased consistently when classifying healthy and the respective UPDRS scores. A similar strategy was later designed by Nodehi et al. [9], which found a moderate correlation between reach kinematic measures (i.e., normalized movement time and peak velocity) and manual dexterity. However, in this particular case, the authors did not report extensive details about the software and postprocessing operations. A less recent paper focused on a similar aim (namely, evaluating the kinematics of the reach-to-grasp movements) using, differently, a flex sensor glove to evaluate potential differences between subjects affected by vascular parkinsonism, idiopathic subjects with Parkinson’s disease, and healthy controls [44]. The authors assessed how the “movement time” kinematic feature demonstrated a statistically significant difference between Parkinson’s disease patients and healthy controls, a particular case that could be found also in our findings, which show higher scores for “duration” in subjects with Parkinson’s disease.

Regarding reaching movements and the Hogan model, we recommend the work of Hu et al. [45]. Albeit with a different aim, the authors investigated fast front reaching movements of an upper extremity of both healthy and subjects with Parkinson’s disease using an arm support apparatus (equipped with four magnetic sensors) aiming at collecting kinematic data, used also to compute the indexes of the minimum jerk trajectory model [46], of subjects, after signal processing and filtering (the authors did not provide details in this regard). The obtained findings showed method indexes could evidence differences between pathological subjects and healthy controls both in the absence or in the presence of the surface stimulation of the superficial radial nerve; nevertheless, this strategy did not show a quantitative outcome assessment before and after the experiments [45].

Finally, to the best of the authors’ knowledge, this seems the first study, which proposed the approach (described previously in the paper) to achieve a quantitative rehabilitation outcome for patients with Parkinson’s disease considering mainly features extracted by reaching task submovements. In the literature, indeed, very few publications addressing a similar issue can be found: in particular, regarding reaching tasks submovements, we highlight the contributions of Simo et al. [47] for post-stroke patients and Carpinella et al. for multiple sclerosis subjects [37].

Albeit the promising findings and the successful proof-of-concept, the results are not conclusive. For instance, further experiments are needed due to the relatively small sample size considered, which implies the presented results should be considered as preliminary ones. However, it should be noted that, as also highlighted elsewhere [1], given the nature of the rehabilitation, which should be individualized and vertically focused on the single case, the use of too many large samples could prevent the design of personalized patients’ treatment based on specific clinical information of the subjects involved in the rehabilitation protocol. The authors are also aware that there could be a potential influence of confounding factors related to the population characteristics affecting the capability of the proposed approach to be used in the classification of Parkinson’s disease. However, since this analysis would require a far larger number of subjects, it is out of the scope of this study, which instead aims at presenting the methodological approach to the processing and analysis of kinematic signals and its applicability to distinguish different motion patterns.

### Future Perspectives of the Study

This work introduced a methodological approach to analyze motion signals during a complex reaching task in healthy and Parkinson’s disease individuals and extracted an ensemble of kinematic parameters to study the upper limb control mechanism. Thirteen kinematic features were estimated and compared for each of the eight submovements, and a subset of significant parameters was selected as the most promising for the definition of a potentially reliable indicator of the rehabilitation outcome in Parkinson disease. Given the strong relationships between physical exercises and the management of neurodegenerative symptoms [48,49,50,51], from the clinical point of view, this study could help in the design of novel training tasks and exercises for the rehabilitation of upper limb function aimed at improving the value of those kinematic parameters identified as the most significant ones in this preliminary research. In addition, from the research perspective, the extracted parameters could be used as a basis for a further in-depth investigation of the upper limb control mechanisms in Parkinson’s disease, e.g., for the design of physiological control systems taking into account the kinematic parameters here estimated and selected as well as their correlation with the state of the patients, the stage of the pathology, and other patient-related clinical information. Future studies will aim at expanding the dataset and compute a synthetic index that could serve as a quantitative indicator of the rehabilitation output after upper limb reaching tasks. With the aim of assessing the potential of the proposed methodological approach in the classification of different neurodegenerative disorders and diseases, further works involving a significantly higher number of subjects will be carried out and the presented software will undergo a deeper validation phase. Moreover, an interesting development could be the use of machine learning algorithms to perform classification tasks on the features extracted through our software and/or on signals themselves. Furthermore, co-registration of motion patterns through goniometric and accelerometric sensors together with electromyography and electroencephalography signals is envisaged. Indeed, we plan to conduct further studies aimed at conducting simultaneous kinematic and electromyography analysis in order to investigate relationships between muscles’ electrical activity and motion patterns in healthy and diseased subjects and identify possible correlations between the proposed kinematic features and additional electromyographic parameters.

## 5. Conclusions

In this work, we presented a method for assessing the motor planning and control in both healthy and diseases subjects by means of an upper limb reaching task and estimation of an ensemble of kinematic parameters. The obtained results indicated that the extracted features to discriminate between healthy and Parkinson subjects proved to be statistically significant for each submovement. As expected, pathological subjects displayed a more fragmentary and discontinuous motion, which can be summarized in the averaged values of the estimated kinematic parameters. The parameters with the greatest number of significances across the submovements were duration, mean velocity, maximum velocity, maximum acceleration, and smoothness. These features could be useful also for performing harder clinical tasks such as a differential diagnosis, which is commonly required for studying Parkinson’s.

## Figures and Tables

**Table 1:** Phases of the implemented kinematic task protocol.

ID	Task	Sub-Movement	Description
1	horizontal reaching task	middle to outer right	Extension of the right (left) shoulder from the middle position to the outer right (left) position on the horizontal plane
2	horizontal reaching task	outer right to middle	Flexion of the right (left) shoulder from outer right (left) position to middle position on the horizontal plane
3	vertical reaching task	middle to top	Elevation from the middle position upwards on the sagittal plane
4	vertical reaching task	top to middle	Lowering from the top to the middle position on the sagittal plane
5	horizontal reaching task	middle to outer left	Flexion of the right (left) shoulder from the middle position to the outer left (right) position on the horizontal plane
6	horizontal reaching task	outer left to middle	Extension of the right (left) shoulder from outer left (right) position to middle position on the horizontal plane
7	vertical reaching task	middle to bottom	Lowering from the middle position downwards on the sagittal plane
8	vertical reaching task	bottom to middle	Elevation from the bottom to the middle position on the sagittal plane

**Table 2:** Submovement 1 kinematic parameters’ statistics and statistical tests for comparing groups, Mann-Whitney or *t* test according to the distribution of data (please see Supplementary Table S1 for more details).

Submovement 1 Kinematic Parameters	Class	Descriptive Statistics				Mann-Whitney (*) or *t* (**) Test
		Mean	Standard Deviation	Median	Interquartile Range	*p*-Value ^§^
amplitude	Healthy	29.25	1.815	29.00	2.750	0.007 *
	Parkinson	27.08	2.644	27.00	3.000	
duration	Healthy	1.519	0.151	1.486	0.220	0.002 **
	Parkinson	3.296	1.510	2.745	2.740	
v_mean	Healthy	19.50	2.276	20.00	3.750	0.000 **
	Parkinson	10.00	3.814	10.00	7.250	
v_max	Healthy	35.72	5.066	36.85	7.040	0.000 **
	Parkinson	22.65	7.609	21.43	14.340	
a_max	Healthy	99.34	20.90	107.0	36.310	0.010 *
	Parkinson	67.65	27.07	61.24	45.630	
jerk_max	Healthy	454.70	126.7	488.6	233.120	0.089 **
	Parkinson	349.20	162.0	317.3	204.390	
symmetry	Healthy	−1.276	0.061	−1.290	0.100	0.020 **
	Parkinson	−1.374	0.120	−1.382	0.150	
p_mean	Healthy	106.80	1.922	106.7	3.060	0.582 **
	Parkinson	107.20	1.847	107.3	1.970	
p_root_mean	Healthy	11,411.2	411.8	11,379.4	652.210	0.583 **
	Parkinson	11,502.9	394.6	11,520.2	422.530	
variance	Healthy	112.4	13.66	114.2	17.440	0.000 *
	Parkinson	78.64	15.97	82.97	18.780	
skewness	Healthy	−0.167	0.255	−0.207	0.430	0.000 **
	Parkinson	0.457	0.397	0.436	0.560	
kurtosis	Healthy	1.695	0.161	1.700	0.240	0.002 **
	Parkinson	2.337	0.545	2.251	0.460	
smoothness	Healthy	34.23	0.543	33.95	1.020	0.000 *
	Parkinson	37.31	1.741	36.69	3.010	

^§^*p*-values below the significance level (α = 0.05) are reported in bold.

## Data Availability

The data presented in this study cannot be made publicly available due to the privacy policy.

## Supplementary Materials

The following supporting information can be downloaded at: https://www.mdpi.com/article/10.3390/s22051708/s1, Table S1: Sub-movement 1 kinematic parameters’ statistics; Table S2: Sub-movement 2 kinematic parameters’ statistics; Table S3: Sub-movement 3 kinematic parameters’ statistics; Table S4: Sub-movement 4 kinematic parameters’ statistics; Table S5: Sub-movement 5 kinematic parameters’ statistics; Table S6: Sub-movement 6 kinematic parameters’ statistics; Table S7: Sub-movement 7 kinematic parameters’ statistics; Table S8: Sub-movement 8 kinematic parameters’ statistics; Table S9: Sub-movement 9 kinematic parameters’ statistics.
