# Supplementary material for: Statistical Analysis and Kinematic Assessment of Upper Limb Reaching Task in Parkinson’s Disease
Source: Sensors (Basel). 2022 Feb 22;22(5):1708. doi: 10.3390/s22051708 (PMC8915106; doi:10.3390/s22051708)
Supplement: Supplementary file 1 [file sensors-22-01708-s001.zip › sensors-1558202-supplementary.pdf]

sensors

MDPI

Supplementary Material

Statistical analysis and kinematic assessment of upper limb reaching task in Parkinson's disease

Alfonso Maria Ponsiglione[1,†] and Carlo Ricciardi[1,2,†], Francesco Amato[1], Mario Cesarelli[1,2], Giuseppe Cesarelli[2,3,§,*] and Giovanni D'Addio[2,§]

[1] Department of Electrical Engineering and Information Technology, University of Naples "Federico II", Piazzale Tecchio 80, 80125, Naples, Italy. alfonsomaria.ponsiglione@unina.it, carloricciardi.93@gmail.com, framato@unina.it, cesarell@unina.it
[2] Institute of Care and Scientific Research of Telese of ICS Maugeri SPA SB, Via Bagni Vecchi, 1, 82037 Telese Terme (Benevento), Italy. gianni.daddio@icsmaugeri.it
[3] Department of Chemical, Materials and Production Engineering, University of Naples Federico II, Via Claudio 21, 80125, Naples, Italy. giuseppe.cesarelli@unina.it
* Correspondence: giuseppe.cesarelli@unina.it, Piazzale Tecchio 80, 80125, Naples, Italy.
† These authors (A.M.P. and C.R.) equally contributed to the work.
§ These authors (G.C. and G.D.) equally contributed to the work.

Citation: Ponsiglione, A.M.; Ricciardi, C.; Amato, F.; Cesarelli, M.; Cesarelli, G.; D'Addio, G. Statistical Analysis and Kinematic Assessment of Upper Limb Reaching Task in Parkinson's Disease. *Sensors* **2022**, *22*, 1708. https://doi.org/10.3390/s22051708

Academic Editor: Brett Fling

Received: 30 December 2021
Accepted: 17 February 2022
Published: 22 February 2022

Publisher's Note: MDPI stays neutral with regard to jurisdictional claims in published maps and institutional affiliations.

Copyright: © 2022 by the authors. Submitted for possible open access publication under the terms and conditions of the Creative Commons Attribution (CC BY) license (https://creativecommons.org/licenses/by/4.0/).

Table S1. Sub-movement 1 kinematic parameters' statistics.

	Class	Kolmogorov-Smirnov test				Shapiro-Wilk test			
		Value of the statistic	Degrees of freedom	p-value	Normally distributed?	Value of the statistic	Degrees of freedom	p-value	Normally distributed?
Amplitude	Healthy	0.221	12	0.108	Yes	0.944	12	0.557	Yes
	Parkinson	0.198	12	0.200	Yes	0.848	12	0.035	No
Duration	Healthy	0.166	12	0.200	Yes	0.952	12	0.661	Yes
	Parkinson	0.185	12	0.200	Yes	0.883	12	0.096	Yes
v_mean	Healthy	0.170	12	0.200	Yes	0.906	12	0.190	Yes
	Parkinson	0.118	12	0.200	Yes	0.940	12	0.498	Yes
v_max	Healthy	0.221	12	0.108	Yes	0.901	12	0.163	Yes
	Parkinson	0.156	12	0.200	Yes	0.944	12	0.553	Yes
a_max	Healthy	0.252	12	0.034	No	0.833	12	0.023	No
	Parkinson	0.170	12	0.200	Yes	0.941	12	0.505	Yes
jerk_max	Healthy	0.166	12	0.200	Yes	0.937	12	0.462	Yes
	Parkinson	0.183	12	0.200	Yes	0.892	12	0.126	Yes
Simmetry	Healthy	0.155	12	0.200	Yes	0.966	12	0.863	Yes
	Parkinson	0.167	12	0.200	Yes	0.956	12	0.728	Yes
p_mean	Healthy	0.148	12	0.200	Yes	0.970	12	0.916	Yes
	Parkinson	0.247	12	0.042	No	0.903	12	0.173	Yes
p_root_mean	Healthy	0.151	12	0.200	Yes	0.969	12	0.896	Yes
	Parkinson	0.243	12	0.048	No	0.905	12	0.186	Yes
Variance	Healthy	0.184	12	0.200	Yes	0.933	12	0.416	Yes
	Parkinson	0.205	12	0.174	Yes	0.870	12	0.066	No
Skewness	Healthy	0.127	12	0.200	Yes	0.969	12	0.905	Yes
	Parkinson	0.107	12	0.200	Yes	0.969	12	0.895	Yes
Kurtosys	Healthy	0.133	12	0.200	Yes	0.984	12	0.995	Yes
	Parkinson	0.196	12	0.200	Yes	0.920	12	0.290	Yes
Smoothness	Healthy	0.275	12	0.013	No	0.821	12	0.017	No
	Parkinson	0.190	12	0.200	Yes	0.922	12	0.300	Yes

	Class	Kolmogorov-Smirnov test	Shapiro-Wilk test	Descriptive statistics			Levene's test	Student's test	Test mediane (prima riga) / Mann-Withney test (seconda riga)
		Normally distributed?	Normally distributed?	Mean	Standard deviation	Median	p-value	p-value	p-value
**Amplitude**	Healthy	Yes	Yes	29.250	1.815	29.000	0.494	**0.029**	0.036
	Parkinson	Yes	No	27.080	2.644	27.000		0.030	**0.007**
**Duration**	Healthy	Yes	Yes	1.519	0.151	1.486	0.000	0.001	0.000
	Parkinson	Yes	Yes	3.296	1.510	2.745		**0.002**	**0.000**
**v_mean**	Healthy	Yes	Yes	19.500	2.276	20.000	0.060	**0.000**	0.000
	Parkinson	Yes	Yes	10.000	3.814	10.000		0.000	**0.000**
**v_max**	Healthy	Yes	Yes	35.720	5.066	36.850	0.086	**0.000**	0.003
	Parkinson	Yes	Yes	22.650	7.609	21.430		0.000	**0.000**
**a_max**	Healthy	No	No	99.340	20.900	107.000	0.366	**0.004**	0.039
	Parkinson	Yes	Yes	67.650	27.070	61.240		0.004	**0.010**
jerk_max	Healthy	Yes	Yes	454.700	126.700	488.600	0.566	**0.089**	0.220
	Parkinson	Yes	Yes	349.200	162.000	317.300		0.090	0.114
**Simmetry**	Healthy	Yes	Yes	-1.276	0.061	-1.290	0.121	**0.020**	0.220
	Parkinson	Yes	Yes	-1.374	0.120	-1.382		0.023	**0.028**
p_mean	Healthy	Yes	Yes	106.800	1.922	106.700	0.527	**0.582**	0.220
	Parkinson	No	Yes	107.200	1.847	107.300		0.582	0.378
p_root_mean	Healthy	Yes	Yes	11411.200	411.800	11379.400	0.523	**0.583**	0.220
	Parkinson	No	Yes	11502.900	394.600	11520.200		0.583	0.378
**Variance**	Healthy	Yes	Yes	112.400	13.660	114.200	0.567	**0.000**	0.000
	Parkinson	Yes	No	78.640	15.970	82.970		0.000	**0.000**
**Skewness**	Healthy	Yes	Yes	-0.167	0.255	-0.207	0.133	**0.000**	0.003
	Parkinson	Yes	Yes	0.457	0.397	0.436		0.000	**0.000**
**Kurtosys**	Healthy	Yes	Yes	1.695	0.161	1.700	0.046	0.001	0.000
	Parkinson	Yes	Yes	2.337	0.545	2.251		**0.002**	**0.000**
**Smoothness**	Healthy	No	No	34.230	0.543	33.950	0.001	0.000	0.000
	Parkinson	Yes	Yes	37.310	1.741	36.690		**0.000**	**0.000**

Table S2. Sub-movement 2 kinematic parameters' statistics.

	Class	Kolmogorov-Smirnov test				Shapiro-Wilk test			
		Value of the statistic	Degrees of freedom	p-value	Normally distributed?	Value of the statistic	Degrees of freedom	p-value	Normally distributed?
Amplitude	Healthy	0.225	12	0.095	YES	0.903	12	0.173	YES
	Parkinson	0.277	12	0.012	NO	0.871	12	0.067	YES
Duration	Healthy	0.194	12	0.200	YES	0.832	12	0.022	NO
	Parkinson	0.272	12	0.014	NO	0.854	12	0.041	NO
v_mean	Healthy	0.222	12	0.106	YES	0.850	12	0.037	NO
	Parkinson	0.274	12	0.013	NO	0.721	12	0.001	NO
v_max	Healthy	0.146	12	0.200	YES	0.950	12	0.636	YES
	Parkinson	0.250	12	0.037	NO	0.733	12	0.002	NO
a_max	Healthy	0.160	12	0.200	YES	0.915	12	0.249	YES
	Parkinson	0.277	12	0.012	NO	0.746	12	0.002	NO
jerk_max	Healthy	0.165	12	0.200	YES	0.940	12	0.499	YES
	Parkinson	0.331	12	0.001	NO	0.714	12	0.001	NO
Simmetry	Healthy	0.170	12	0.200	YES	0.948	12	0.601	YES
	Parkinson	0.280	12	0.010	NO	0.685	12	0.001	NO
p_mean	Healthy	0.144	12	0.200	YES	0.946	12	0.573	YES
	Parkinson	0.165	12	0.200	YES	0.942	12	0.523	YES
p_root_mean	Healthy	0.141	12	0.200	YES	0.945	12	0.566	YES
	Parkinson	0.157	12	0.200	YES	0.943	12	0.532	YES
Variance	Healthy	0.135	12	0.200	YES	0.934	12	0.420	YES
	Parkinson	0.339	12	0.000	NO	0.642	12	0.000	NO
Skewness	Healthy	0.089	12	0.200	YES	0.993	12	1.000	YES
	Parkinson	0.201	12	0.196	YES	0.896	12	0.140	YES
Kurtosys	Healthy	0.231	12	0.075	YES	0.768	12	0.004	NO
	Parkinson	0.274	12	0.013	NO	0.728	12	0.002	NO
Smoothness	Healthy	0.159	12	0.200	YES	0.893	12	0.130	YES
	Parkinson	0.195	12	0.200	YES	0.865	12	0.056	YES

	Class	Kolmogorov-Smirnov test	Shapiro-Wilk test	Descriptive statistics			Levene's test	Student's test	Test mediane (prima riga) / Mann-Withney test (seconda riga)
		Normally distributed?	Normally distributed?	Mean	Standard deviation	Median	p-value	p-value	p-value
Amplitude	Healthy	YES	YES	-30.583	2.843	-31.000	0.135	**0.444**	0.220
	Parkinson	NO	YES	-29.333	4.774	-28.000		0.446	0.219
**Duration**	Healthy	YES	NO	1.492	0.267	1.444	0.001	0.004	0.003
	Parkinson	NO	NO	3.165	1.765	2.617		**0.007**	**0.001**
**v_mean**	Healthy	YES	NO	-21.083	3.825	-21.500	0.169	**0.009**	0.000
	Parkinson	NO	NO	-12.583	9.472	-10.500		0.012	**0.001**
**v_max**	Healthy	YES	YES	-44.257	5.355	-44.807	0.143	**0.004**	0.000
	Parkinson	NO	NO	-28.000	16.814	-25.262		0.007	**0.000**
**a_max**	Healthy	YES	YES	127.339	21.641	129.427	0.116	**0.008**	0.039
	Parkinson	NO	NO	76.999	55.731	57.564		0.011	**0.002**
**jerk_max**	Healthy	YES	YES	658.786	140.284	635.824	0.100	**0.028**	0.039
	Parkinson	NO	NO	405.260	346.466	293.945		0.033	**0.006**
Simmetry	Healthy	YES	YES	-1.195	0.036	-1.203	0.036	0.164	0.684
	Parkinson	NO	NO	-1.276	0.193	-1.212		**0.176**	0.266
p_mean	Healthy	YES	YES	102.337	1.785	102.627	0.084	**0.277**	0.684
	Parkinson	YES	YES	100.943	3.944	101.570		0.282	0.291
p_root_mean	Healthy	YES	YES	10475.764	366.098	10532.248	0.095	**0.294**	0.684
	Parkinson	YES	YES	10203.702	796.180	10316.554		0.299	0.291
**Variance**	Healthy	YES	YES	126.255	22.139	129.616	0.387	**0.071**	0.003
	Parkinson	NO	NO	98.447	45.797	88.923		0.077	**0.002**
Skewness	Healthy	YES	YES	-0.177	0.388	-0.139	0.120	**0.869**	0.684
	Parkinson	YES	YES	-0.221	0.814	-0.218		0.869	0.843
Kurtosys	Healthy	YES	NO	1.754	0.252	1.677	0.007	0.062	0.220
	Parkinson	NO	NO	2.460	1.218	2.043		**0.073**	0.089
**Smoothness**	Healthy	YES	YES	34.705	0.987	34.501	0.009	0.008	0.003
	Parkinson	YES	YES	37.011	2.550	36.019		**0.011**	**0.005**

Table S3. Sub-movement 3 kinematic parameters' statistics.

	Class	Kolmogorov-Smirnov test				Shapiro-Wilk test			
		Value of the statistic	Degrees of freedom	p-value	Normally distributed?	Value of the statistic	Degrees of freedom	p-value	Normally distributed?
Amplitude	Healthy	0.230	12	0.079	YES	0.894	12	0.132	YES
	Parkinson	0.190	12	0.200	YES	0.932	12	0.401	YES
Duration	Healthy	0.337	12	0.001	NO	0.588	12	0.000	NO
	Parkinson	0.321	12	0.001	NO	0.763	12	0.004	NO
v_mean	Healthy	0.200	12	0.200	YES	0.910	12	0.213	YES
	Parkinson	0.155	12	0.200	YES	0.925	12	0.333	YES
v_max	Healthy	0.138	12	0.200	YES	0.938	12	0.476	YES
	Parkinson	0.144	12	0.200	YES	0.956	12	0.729	YES
a_max	Healthy	0.115	12	0.200	YES	0.962	12	0.806	YES
	Parkinson	0.163	12	0.200	YES	0.932	12	0.405	YES
jerk_max	Healthy	0.202	12	0.188	YES	0.943	12	0.534	YES
	Parkinson	0.209	12	0.157	YES	0.907	12	0.197	YES
Simmetry	Healthy	0.260	12	0.025	NO	0.795	12	0.008	NO
	Parkinson	0.240	12	0.055	YES	0.829	12	0.020	NO
p_mean	Healthy	0.206	12	0.169	YES	0.915	12	0.250	YES
	Parkinson	0.204	12	0.180	YES	0.863	12	0.053	YES
p_root_mean	Healthy	0.204	12	0.182	YES	0.918	12	0.269	YES
	Parkinson	0.195	12	0.200	YES	0.878	12	0.082	YES
Variance	Healthy	0.164	12	0.200	YES	0.859	12	0.048	NO
	Parkinson	0.153	12	0.200	YES	0.973	12	0.939	YES
Skewness	Healthy	0.164	12	0.200	YES	0.930	12	0.380	YES
	Parkinson	0.213	12	0.140	YES	0.847	12	0.034	NO
Kurtosys	Healthy	0.162	12	0.200	YES	0.937	12	0.460	YES
	Parkinson	0.335	12	0.001	NO	0.611	12	0.000	NO
Smoothness	Healthy	0.216	12	0.128	YES	0.777	12	0.005	NO
	Parkinson	0.272	12	0.014	NO	0.831	12	0.022	NO

	Class	Kolmogorov-Smirnov test	Shapiro-Wilk test	Descriptive statistics			Levene's test	Student's test	Test mediane (prima riga) / Mann-Withney test (seconda riga)
		Normally distributed?	Normally distributed?	Mean	Standard deviation	Median	p-value	p-value	p-value
Amplitude	Healthy	YES	YES	-29.917	1.676	-30.000	0.350	**0.201**	0.371
	Parkinson	YES	YES	-31.167	2.823	-31.000		0.204	0.114
**Duration**	Healthy	NO	NO	1.689	0.833	1.400	0.208	**0.009**	0.000
	Parkinson	NO	NO	3.000	1.360	2.622		0.011	**0.000**
**v_mean**	Healthy	YES	YES	-19.833	5.374	-20.500	0.689	**0.001**	0.003
	Parkinson	YES	YES	-12.000	4.156	-12.500		0.001	**0.001**
**v_max**	Healthy	YES	YES	-39.297	8.901	-40.784	0.829	**0.001**	0.003
	Parkinson	YES	YES	-26.504	8.051	-25.957		0.001	**0.001**
**a_max**	Healthy	YES	YES	108.529	27.721	110.480	0.678	**0.001**	0.039
	Parkinson	YES	YES	68.795	25.189	70.853		0.001	**0.001**
**jerk_max**	Healthy	YES	YES	525.353	152.732	502.547	0.920	**0.007**	0.220
	Parkinson	YES	YES	349.138	138.459	301.579		0.007	**0.008**
Simmetry	Healthy	NO	NO	-1.116	0.043	-1.096	0.554	**0.845**	0.684
	Parkinson	YES	NO	-1.119	0.036	-1.104		0.845	0.443
p_mean	Healthy	YES	YES	72.175	1.564	72.772	0.312	**0.529**	0.684
	Parkinson	YES	YES	71.604	2.667	72.033		0.530	0.713
p_root_mean	Healthy	YES	YES	5211.429	224.427	5295.705	0.312	**0.542**	0.684
	Parkinson	YES	YES	5133.597	372.642	5189.044		0.543	0.713
Variance	Healthy	YES	NO	117.922	15.926	114.285	0.232	**0.804**	1.000
	Parkinson	YES	YES	115.646	27.125	116.681		0.805	0.932
Skewness	Healthy	YES	YES	-0.061	0.262	-0.036	0.526	**0.027**	0.684
	Parkinson	YES	NO	-0.386	0.396	-0.265		0.029	0.060
Kurtosys	Healthy	YES	YES	1.670	0.124	1.633	0.051	**0.171**	0.220
	Parkinson	NO	NO	2.089	1.015	1.798		0.184	0.347
**Smoothness**	Healthy	YES	NO	34.722	1.556	34.140	0.660	**0.010**	0.003
	Parkinson	NO	NO	36.677	1.843	36.203		0.010	**0.002**

Table S4. Sub-movement 4 kinematic parameters' statistics.

	Class	Kolmogorov-Smirnov test				Shapiro-Wilk test			
		Value of the statistic	Degrees of freedom	p-value	Normally distributed?	Value of the statistic	Degrees of freedom	p-value	Normally distributed?
Amplitude	Healthy	0.188	12	0.200	YES	0.957	12	0.734	YES
	Parkinson	0.178	12	0.200	YES	0.928	12	0.355	YES
Duration	Healthy	0.287	12	0.007	NO	0.740	12	0.002	NO
	Parkinson	0.283	12	0.009	NO	0.720	12	0.001	NO
v_mean	Healthy	0.126	12	0.200	YES	0.938	12	0.468	YES
	Parkinson	0.169	12	0.200	YES	0.963	12	0.819	YES
v_max	Healthy	0.157	12	0.200	YES	0.964	12	0.835	YES
	Parkinson	0.246	12	0.043	NO	0.881	12	0.091	YES
a_max	Healthy	0.135	12	0.200	YES	0.965	12	0.848	YES
	Parkinson	0.184	12	0.200	YES	0.949	12	0.624	YES
jerk_max	Healthy	0.198	12	0.200	YES	0.914	12	0.237	YES
	Parkinson	0.202	12	0.190	YES	0.958	12	0.748	YES
Simmetry	Healthy	0.107	12	0.200	YES	0.978	12	0.975	YES
	Parkinson	0.240	12	0.054	YES	0.795	12	0.008	NO
p_mean	Healthy	0.248	12	0.040	NO	0.868	12	0.061	YES
	Parkinson	0.120	12	0.200	YES	0.934	12	0.426	YES
p_root_mean	Healthy	0.253	12	0.033	NO	0.863	12	0.053	YES
	Parkinson	0.114	12	0.200	YES	0.941	12	0.514	YES
Variance	Healthy	0.098	12	0.200	YES	0.984	12	0.995	YES
	Parkinson	0.222	12	0.107	YES	0.862	12	0.052	YES
Skewness	Healthy	0.365	12	0.000	NO	0.724	12	0.001	NO
	Parkinson	0.182	12	0.200	YES	0.936	12	0.448	YES
Kurtosys	Healthy	0.262	12	0.022	NO	0.674	12	0.000	NO
	Parkinson	0.107	12	0.200	YES	0.973	12	0.938	YES
Smoothness	Healthy	0.275	12	0.013	NO	0.729	12	0.002	NO
	Parkinson	0.176	12	0.200	YES	0.931	12	0.392	YES

	Class	Kolmogorov-Smirnov test	Shapiro-Wilk test	Descriptive statistics			Levene's test	Student's test	Test mediane (prima riga) / Mann-Withney test (seconda riga)
		Normally distributed?	Normally distributed?	Mean	Standard deviation	Median	p-value	p-value	p-value
Amplitude	Healthy	YES	YES	31.000	2.045	30.500	0.062	**0.556**	0.680
	Parkinson	YES	YES	29.917	5.931	29.500		0.560	0.319
**Duration**	Healthy	NO	NO	1.457	0.596	1.324	0.259	**0.009**	0.003
	Parkinson	NO	NO	2.634	1.294	2.479		0.012	**0.001**
**v_mean**	Healthy	YES	YES	23.500	6.802	24.000	0.593	**0.001**	0.009
	Parkinson	YES	YES	13.417	5.534	13.000		0.001	**0.001**
**v_max**	Healthy	YES	YES	45.898	10.213	44.818	0.643	**0.006**	0.039
	Parkinson	NO	YES	33.092	10.280	34.205		0.006	**0.007**
**a_max**	Healthy	YES	YES	133.435	34.096	129.152	0.775	**0.006**	0.039
	Parkinson	YES	YES	90.548	34.477	92.995		0.006	**0.005**
**jerk_max**	Healthy	YES	YES	711.631	275.326	615.263	0.386	**0.029**	0.039
	Parkinson	YES	YES	469.188	230.619	466.348		0.029	**0.017**
Simmetry	Healthy	YES	YES	-1.085	0.019	-1.087	0.334	**0.536**	1.000
	Parkinson	YES	NO	-1.093	0.037	-1.087		0.539	0.977
**p_mean**	Healthy	NO	YES	76.345	1.766	76.241	0.292	**0.032**	0.039
	Parkinson	YES	YES	78.364	2.495	78.358		0.033	**0.020**
**p_root_mean**	Healthy	NO	YES	5831.480	272.332	5812.679	0.270	**0.031**	0.039
	Parkinson	YES	YES	6146.606	386.202	6139.987		0.032	**0.020**
Variance	Healthy	YES	YES	127.798	20.055	128.359	0.104	**0.343**	0.220
	Parkinson	YES	YES	112.083	52.442	104.063		0.349	0.089
**Skewness**	Healthy	NO	NO	0.042	0.324	-0.034	0.493	**0.005**	0.003
	Parkinson	YES	YES	0.520	0.414	0.555		0.005	**0.008**
**Kurtosys**	Healthy	NO	NO	1.719	0.283	1.638	0.284	**0.002**	0.003
	Parkinson	YES	YES	2.179	0.343	2.160		0.002	**0.001**
**Smoothness**	Healthy	NO	NO	34.380	1.125	34.037	0.388	**0.001**	0.003
	Parkinson	YES	YES	36.505	1.479	36.451		0.001	**0.001**

Table S5. Sub-movement 5 kinematic parameters' statistics.

	Class	Kolmogorov-Smirnov test				Shapiro-Wilk test			
		Value of the statistic	Degrees of freedom	p-value	Normally distributed?	Value of the statistic	Degrees of freedom	p-value	Normally distributed?
Amplitude	Healthy	0.153	12	0.200	YES	0.931	12	0.394	YES
	Parkinson	0.176	12	0.200	YES	0.929	12	0.368	YES
Duration	Healthy	0.110	12	0.200	YES	0.959	12	0.766	YES
	Parkinson	0.165	12	0.200	YES	0.902	12	0.170	YES
v_mean	Healthy	0.207	12	0.163	YES	0.911	12	0.222	YES
	Parkinson	0.165	12	0.200	YES	0.942	12	0.525	YES
v_max	Healthy	0.188	12	0.200	YES	0.896	12	0.141	YES
	Parkinson	0.143	12	0.200	YES	0.949	12	0.618	YES
a_max	Healthy	0.162	12	0.200	YES	0.896	12	0.139	YES
	Parkinson	0.147	12	0.200	YES	0.911	12	0.217	YES
jerk_max	Healthy	0.263	12	0.021	NO	0.834	12	0.023	NO
	Parkinson	0.169	12	0.200	YES	0.869	12	0.063	YES
Simmetry	Healthy	0.372	12	0.000	NO	0.693	12	0.001	NO
	Parkinson	0.380	12	0.000	NO	0.716	12	0.001	NO
p_mean	Healthy	0.204	12	0.180	YES	0.862	12	0.052	YES
	Parkinson	0.149	12	0.200	YES	0.935	12	0.431	YES
p_root_mean	Healthy	0.222	12	0.106	YES	0.840	12	0.027	NO
	Parkinson	0.170	12	0.200	YES	0.943	12	0.544	YES
Variance	Healthy	0.122	12	0.200	YES	0.960	12	0.783	YES
	Parkinson	0.208	12	0.162	YES	0.894	12	0.133	YES
Skewness	Healthy	0.110	12	0.200	YES	0.970	12	0.910	YES
	Parkinson	0.190	12	0.200	YES	0.948	12	0.608	YES
Kurtosys	Healthy	0.232	12	0.075	YES	0.886	12	0.105	YES
	Parkinson	0.262	12	0.022	NO	0.805	12	0.011	NO
Smoothness	Healthy	0.178	12	0.200	YES	0.967	12	0.873	YES
	Parkinson	0.158	12	0.200	YES	0.905	12	0.185	YES

	Class	Kolmogorov-Smirnov test	Shapiro-Wilk test	Descriptive statistics			Levene's test	Student's test	Test mediane (prima riga) / Mann-Withney test (seconda riga)
		Normally distributed?	Normally distributed?	Mean	Standard deviation	Median	p-value	p-value	p-value
Amplitude	Healthy	YES	YES	29.917	2.314	30.000	0.602	**0.815**	0.684
	Parkinson	YES	YES	29.667	2.839	29.000		0.815	0.713
**Duration**	Healthy	YES	YES	1.974	0.291	1.990	0.009	0.000	0.000
	Parkinson	YES	YES	3.166	0.923	3.196		**0.001**	**0.000**
**v_mean**	Healthy	YES	YES	15.250	2.340	15.000	0.398	**0.000**	0.012
	Parkinson	YES	YES	9.833	2.949	9.500		0.000	**0.000**
**v_max**	Healthy	YES	YES	26.449	5.289	26.922	0.582	**0.001**	0.039
	Parkinson	YES	YES	17.718	6.013	17.787		0.001	**0.002**
a_max	Healthy	YES	YES	66.570	15.393	63.058	0.115	**0.048**	0.684
	Parkinson	YES	YES	50.107	22.423	48.349		0.049	0.089
jerk_max	Healthy	NO	NO	314.015	112.669	312.948	0.362	**0.232**	0.684
	Parkinson	YES	YES	249.561	142.699	211.238		0.233	0.143
Simmetry	Healthy	NO	NO	-2.443	6.552	-2.827	0.539	**0.896**	1.000
	Parkinson	NO	NO	-2.927	10.788	-3.219		0.896	0.713
p_mean	Healthy	YES	YES	15.915	1.351	15.470	0.029	0.500	0.684
	Parkinson	YES	YES	15.206	3.317	14.664		**0.503**	0.319
p_root_mean	Healthy	YES	NO	254.976	44.865	239.319	0.020	0.663	0.684
	Parkinson	YES	YES	241.307	97.173	215.077		**0.664**	0.319
Variance	Healthy	YES	YES	110.819	17.970	111.034	0.725	**0.090**	0.220
	Parkinson	YES	YES	96.661	21.000	94.735		0.090	0.068
Skewness	Healthy	YES	YES	-0.391	0.394	-0.415	0.163	**0.563**	0.684
	Parkinson	YES	YES	-0.521	0.661	-0.324		0.564	0.887
**Kurtosys**	Healthy	YES	YES	1.982	0.332	1.835	0.045	0.027	0.220
	Parkinson	NO	NO	2.877	1.269	2.583		**0.035**	**0.014**
**Smoothness**	Healthy	YES	YES	34.750	0.661	34.780	0.272	**0.000**	0.000
	Parkinson	YES	YES	36.486	1.007	36.367		0.000	**0.000**

Table S6. Sub-movement 6 kinematic parameters' statistics.

	Class	Kolmogorov-Smirnov test				Shapiro-Wilk test			
		Value of the statistic	Degrees of freedom	p-value	Normally distributed?	Value of the statistic	Degrees of freedom	p-value	Normally distributed?
Amplitude	Healthy	0.153	12	0.200	YES	0.931	12	0.394	YES
	Parkinson	0.176	12	0.200	YES	0.929	12	0.368	YES
Duration	Healthy	0.110	12	0.200	YES	0.959	12	0.766	YES
	Parkinson	0.165	12	0.200	YES	0.902	12	0.170	YES
v_mean	Healthy	0.207	12	0.163	YES	0.911	12	0.222	YES
	Parkinson	0.165	12	0.200	YES	0.942	12	0.525	YES
v_max	Healthy	0.188	12	0.200	YES	0.896	12	0.141	YES
	Parkinson	0.143	12	0.200	YES	0.949	12	0.618	YES
a_max	Healthy	0.162	12	0.200	YES	0.896	12	0.139	YES
	Parkinson	0.147	12	0.200	YES	0.911	12	0.217	YES
jerk_max	Healthy	0.263	12	0.021	NO	0.834	12	0.023	NO
	Parkinson	0.169	12	0.200	YES	0.869	12	0.063	YES
Simmetry	Healthy	0.372	12	0.000	NO	0.693	12	0.001	NO
	Parkinson	0.380	12	0.000	NO	0.716	12	0.001	NO
p_mean	Healthy	0.204	12	0.180	YES	0.862	12	0.052	YES
	Parkinson	0.149	12	0.200	YES	0.935	12	0.431	YES
p_root_mean	Healthy	0.222	12	0.106	YES	0.840	12	0.027	NO
	Parkinson	0.170	12	0.200	YES	0.943	12	0.544	YES
Variance	Healthy	0.122	12	0.200	YES	0.960	12	0.783	YES
	Parkinson	0.208	12	0.162	YES	0.894	12	0.133	YES
Skewness	Healthy	0.110	12	0.200	YES	0.970	12	0.910	YES
	Parkinson	0.190	12	0.200	YES	0.948	12	0.608	YES
Kurtosys	Healthy	0.232	12	0.075	YES	0.886	12	0.105	YES
	Parkinson	0.262	12	0.022	NO	0.805	12	0.011	NO
Smoothness	Healthy	0.178	12	0.200	YES	0.967	12	0.873	YES
	Parkinson	0.158	12	0.200	YES	0.905	12	0.185	YES

	Class	Kolmogorov-Smirnov test	Shapiro-Wilk test	Descriptive statistics			Levene's test	Student's test	Test mediane (prima riga) / Mann-Withney test (seconda riga)
		Normally distributed?	Normally distributed?	Mean	Standard deviation	Median	p-value	p-value	p-value
Amplitude	Healthy	YES	YES	29.917	2.314	-31.000	0.602	0.815	0.684
	Parkinson	YES	YES	29.667	2.839	-29.000		0.815	0.713
**Duration**	Healthy	YES	YES	1.974	0.291	1.761	0.009	0.000	0.000
	Parkinson	YES	YES	3.166	0.923	3.321		0.001	**0.000**
**v_mean**	Healthy	YES	YES	15.250	2.340	-17.000	0.398	0.000	0.012
	Parkinson	YES	YES	9.833	2.949	-9.000		0.000	**0.000**
**v_max**	Healthy	YES	YES	26.449	5.289	-35.167	0.582	0.001	0.039
	Parkinson	YES	YES	17.718	6.013	-23.620		0.001	**0.002**
a_max	Healthy	YES	YES	66.570	15.393	99.716	0.115	0.048	0.684
	Parkinson	YES	YES	50.107	22.423	60.167		0.049	0.089
jerk_max	Healthy	NO	NO	314.015	112.669	489.102	0.362	0.232	0.684
	Parkinson	YES	YES	249.561	142.699	253.201		0.233	0.143
Simmetry	Healthy	NO	NO	-2.443	6.552	-1.547	0.539	0.896	1.000
	Parkinson	NO	NO	-2.927	10.788	-1.658		0.896	0.713
p_mean	Healthy	YES	YES	15.915	1.351	12.757	0.029	0.500	0.684
	Parkinson	YES	YES	15.206	3.317	12.760		0.503	0.319
p_root_mean	Healthy	YES	NO	254.976	44.865	162.910	0.020	0.663	0.684
	Parkinson	YES	YES	241.307	97.173	162.836		0.664	0.319
Variance	Healthy	YES	YES	110.819	17.970	119.082	0.725	0.090	0.220
	Parkinson	YES	YES	96.661	21.000	100.507		0.090	0.068
Skewness	Healthy	YES	YES	-0.391	0.394	-0.070	0.163	0.563	0.684
	Parkinson	YES	YES	-0.521	0.661	-0.796		0.564	0.887
**Kurtosys**	Healthy	YES	YES	1.982	0.332	2.092	0.045	0.027	0.220
	Parkinson	NO	NO	2.877	1.269	2.334		0.035	**0.014**
**Smoothness**	Healthy	YES	YES	34.750	0.661	34.852	0.272	0.000	0.000
	Parkinson	YES	YES	36.486	1.007	37.407		0.000	**0.000**

Table S7. Sub-movement 7 kinematic parameters' statistics.

	Class	Kolmogorov-Smirnov test				Shapiro-Wilk test			
		Value of the statistic	Degrees of freedom	p-value	Normally distributed?	Value of the statistic	Degrees of freedom	p-value	Normally distributed?
Amplitude	Healthy	0.226	12	0.091	YES	0.913	12	0.231	YES
	Parkinson	0.164	12	0.200	YES	0.945	12	0.572	YES
Duration	Healthy	0.236	12	0.064	YES	0.865	12	0.056	YES
	Parkinson	0.200	12	0.200	YES	0.900	12	0.157	YES
v_mean	Healthy	0.138	12	0.200	YES	0.956	12	0.730	YES
	Parkinson	0.197	12	0.200	YES	0.926	12	0.340	YES
v_max	Healthy	0.154	12	0.200	YES	0.926	12	0.340	YES
	Parkinson	0.167	12	0.200	YES	0.940	12	0.504	YES
a_max	Healthy	0.178	12	0.200	YES	0.897	12	0.146	YES
	Parkinson	0.189	12	0.200	YES	0.931	12	0.392	YES
jerk_max	Healthy	0.158	12	0.200	YES	0.909	12	0.209	YES
	Parkinson	0.258	12	0.027	NO	0.842	12	0.029	NO
Simmetry	Healthy	0.154	12	0.200	YES	0.941	12	0.507	YES
	Parkinson	0.153	12	0.200	YES	0.954	12	0.702	YES
p_mean	Healthy	0.193	12	0.200	YES	0.924	12	0.325	YES
	Parkinson	0.114	12	0.200	YES	0.968	12	0.893	YES
p_root_mean	Healthy	0.177	12	0.200	YES	0.933	12	0.415	YES
	Parkinson	0.136	12	0.200	YES	0.968	12	0.891	YES
Variance	Healthy	0.187	12	0.200	YES	0.940	12	0.502	YES
	Parkinson	0.178	12	0.200	YES	0.882	12	0.092	YES
Skewness	Healthy	0.114	12	0.200	YES	0.948	12	0.602	YES
	Parkinson	0.260	12	0.025	NO	0.841	12	0.029	NO
Kurtosys	Healthy	0.206	12	0.168	YES	0.923	12	0.314	YES
	Parkinson	0.282	12	0.009	NO	0.783	12	0.006	NO
Smoothness	Healthy	0.107	12	0.200	YES	0.983	12	0.994	YES
	Parkinson	0.142	12	0.200	YES	0.949	12	0.618	YES

	Class	Kolmogorov-Smirnov test	Shapiro-Wilk test	Descriptive statistics			Levene's test	Student's test	Test mediane (prima riga) / Mann-Withney test (seconda riga)
		Normally distributed?	Normally distributed?	Mean	Standard deviation	Median	p-value	p-value	p-value
Amplitude	Healthy	YES	YES	-29.333	2.229	-29.000	0.253	**0.048**	0.414
	Parkinson	YES	YES	-27.250	2.633	-27.500		0.049	0.114
**Duration**	Healthy	YES	YES	2.344	0.623	2.142	0.000	0.000	0.000
	Parkinson	YES	YES	5.453	1.962	4.790		**0.000**	**0.000**
**v_mean**	Healthy	YES	YES	-13.250	3.441	-13.500	0.343	**0.000**	0.000
	Parkinson	YES	YES	-5.750	2.261	-5.500		0.000	**0.000**
**v_max**	Healthy	YES	YES	-26.572	6.844	-27.933	0.910	**0.000**	0.003
	Parkinson	YES	YES	-15.152	6.291	-14.860		0.000	**0.000**
**a_max**	Healthy	YES	YES	72.973	17.014	73.129	0.392	**0.001**	0.003
	Parkinson	YES	YES	43.578	21.454	36.528		0.001	**0.002**
**jerk_max**	Healthy	YES	YES	368.106	126.605	348.809	0.813	**0.044**	0.003
	Parkinson	NO	NO	251.675	139.856	217.985		0.044	**0.024**
**Simmetry**	Healthy	YES	YES	-1.248	0.068	-1.233	0.061	**0.009**	0.220
	Parkinson	YES	YES	-1.360	0.118	-1.337		0.011	**0.014**
p_mean	Healthy	YES	YES	-15.992	1.908	-16.594	0.091	**0.210**	0.684
	Parkinson	YES	YES	-14.575	3.284	-14.440		0.213	0.378
p_root_mean	Healthy	YES	YES	259.078	59.489	275.356	0.129	**0.263**	0.684
	Parkinson	YES	YES	222.307	93.545	208.968		0.265	0.378
Variance	Healthy	YES	YES	100.996	19.564	95.634	0.123	**0.094**	0.220
	Parkinson	YES	YES	83.587	28.392	79.252		0.096	0.101
Skewness	Healthy	YES	YES	-0.031	0.419	-0.073	0.089	**0.029**	0.220
	Parkinson	NO	NO	-0.552	0.651	-0.311		0.031	0.078
Kurtosys	Healthy	YES	YES	2.112	0.335	2.235	0.000	0.056	1.000
	Parkinson	NO	NO	3.062	1.597	2.225		**0.067**	0.319
**Smoothness**	Healthy	YES	YES	35.812	1.250	35.810	0.067	**0.000**	0.003
	Parkinson	YES	YES	39.543	1.814	39.459		0.000	**0.000**

Table S8. Sub-movement 8 kinematic parameters' statistics.

	Class	Kolmogorov-Smirnov test				Shapiro-Wilk test			
		Value of the statistic	Degrees of freedom	p-value	Normally distributed?	Value of the statistic	Degrees of freedom	p-value	Normally distributed?
Amplitude	Healthy	0.286	12	0.008	NO	0.711	12	0.001	NO
	Parkinson	0.145	12	0.200	YES	0.934	12	0.421	YES
Duration	Healthy	0.257	12	0.028	NO	0.918	12	0.267	YES
	Parkinson	0.194	12	0.200	YES	0.904	12	0.180	YES
v_mean	Healthy	0.238	12	0.059	YES	0.792	12	0.008	NO
	Parkinson	0.181	12	0.200	YES	0.952	12	0.663	YES
v_max	Healthy	0.182	12	0.200	YES	0.915	12	0.249	YES
	Parkinson	0.115	12	0.200	YES	0.956	12	0.722	YES
a_max	Healthy	0.165	12	0.200	YES	0.923	12	0.307	YES
	Parkinson	0.132	12	0.200	YES	0.943	12	0.537	YES
jerk_max	Healthy	0.174	12	0.200	YES	0.888	12	0.111	YES
	Parkinson	0.186	12	0.200	YES	0.899	12	0.155	YES
Simmetry	Healthy	0.154	12	0.200	YES	0.972	12	0.929	YES
	Parkinson	0.246	12	0.043	NO	0.824	12	0.018	NO
p_mean	Healthy	0.199	12	0.200	YES	0.925	12	0.328	YES
	Parkinson	0.184	12	0.200	YES	0.951	12	0.650	YES
p_root_mean	Healthy	0.218	12	0.119	YES	0.890	12	0.117	YES
	Parkinson	0.216	12	0.128	YES	0.933	12	0.414	YES
Variance	Healthy	0.257	12	0.028	NO	0.765	12	0.004	NO
	Parkinson	0.261	12	0.023	NO	0.817	12	0.015	NO
Skewness	Healthy	0.246	12	0.043	NO	0.878	12	0.082	YES
	Parkinson	0.118	12	0.200	YES	0.983	12	0.994	YES
Kurtosys	Healthy	0.247	12	0.041	NO	0.854	12	0.041	NO
	Parkinson	0.224	12	0.100	YES	0.862	12	0.052	YES
Smoothness	Healthy	0.189	12	0.200	YES	0.887	12	0.109	YES
	Parkinson	0.204	12	0.182	YES	0.917	12	0.260	YES

	Class	Kolmogorov-Smirnov test	Shapiro-Wilk test	Descriptive statistics			Levene's test	Student's test	Test mediane (prima riga) / Mann-Withney test (seconda riga)
		Normally distributed?	Normally distributed?	Mean	Standard deviation	Median	p-value	p-value	p-value
**Amplitude**	Healthy	NO	NO	28.667	3.676	29.000	0.438	**0.071**	0.039
	Parkinson	YES	YES	25.917	3.423	25.500		0.071	**0.045**
**Duration**	Healthy	NO	YES	1.698	0.350	1.732	0.007	0.000	0.000
	Parkinson	YES	YES	3.176	0.872	2.976		**0.000**	**0.000**
**v_mean**	Healthy	YES	NO	17.833	5.458	17.000	0.305	**0.000**	0.000
	Parkinson	YES	YES	8.667	2.708	8.000		0.000	**0.000**
**v_max**	Healthy	YES	YES	36.169	9.403	36.578	0.225	**0.000**	0.003
	Parkinson	YES	YES	19.845	5.275	20.096		0.000	**0.000**
**a_max**	Healthy	YES	YES	98.198	31.739	99.334	0.211	**0.000**	0.039
	Parkinson	YES	YES	52.913	18.647	55.154		0.000	**0.000**
**jerk_max**	Healthy	YES	YES	505.417	228.150	464.258	0.329	**0.004**	0.039
	Parkinson	YES	YES	258.993	131.020	241.949		0.005	**0.003**
Simmetry	Healthy	YES	YES	-1.142	0.039	-1.149	0.869	**0.443**	1.000
	Parkinson	NO	NO	-1.155	0.040	-1.146		0.443	0.755
p_mean	Healthy	YES	YES	-13.244	2.437	-12.437	0.401	**0.931**	1.000
	Parkinson	YES	YES	-13.149	2.850	-12.319		0.931	0.932
p_root_mean	Healthy	YES	YES	180.838	69.117	154.675	0.462	**0.987**	1.000
	Parkinson	YES	YES	180.333	76.650	151.775		0.987	0.932
**Variance**	Healthy	NO	NO	106.236	23.488	108.718	0.372	**0.042**	0.039
	Parkinson	NO	NO	84.041	26.677	71.620		0.042	**0.039**
Skewness	Healthy	NO	YES	0.197	0.422	0.412	0.783	**0.852**	0.684
	Parkinson	YES	YES	0.232	0.468	0.227		0.852	0.932
Kurtosys	Healthy	NO	NO	1.837	0.176	1.761	0.042	0.176	0.684
	Parkinson	YES	YES	2.059	0.521	2.081		**0.185**	0.347
**Smoothness**	Healthy	YES	YES	34.853	0.877	35.083	0.717	**0.000**	0.000
	Parkinson	YES	YES	36.863	0.939	36.900		0.000	**0.000**

Table S9. Average parameters' statistics.

	Class	Kolmogorov-Smirnov test				Shapiro-Wilk test			
		Value of the statistic	Degrees of freedom	p-value	Normally distributed?	Value of the statistic	Degrees of freedom	p-value	Normally distributed?
Amplitude	Healthy	0.314	12	0.002	NO	0.675	12	0.000	NO
	Parkinson	0.251	12	0.036	NO	0.848	12	0.035	NO
Duration	Healthy	0.131	12	0.200	YES	0.949	12	0.627	YES
	Parkinson	0.180	12	0.200	YES	0.921	12	0.293	YES
v_mean	Healthy	0.156	12	0.200	YES	0.934	12	0.423	YES
	Parkinson	0.229	12	0.083	YES	0.918	12	0.267	YES
v_max	Healthy	0.278	12	0.011	NO	0.785	12	0.006	NO
	Parkinson	0.167	12	0.200	YES	0.940	12	0.499	YES
a_max	Healthy	0.154	12	0.200	YES	0.968	12	0.885	YES
	Parkinson	0.220	12	0.113	YES	0.863	12	0.053	YES
jerk_max	Healthy	0.341	12	0.000	NO	0.714	12	0.001	NO
	Parkinson	0.375	12	0.000	NO	0.722	12	0.001	NO
Simmetry	Healthy	0.236	12	0.064	YES	0.886	12	0.106	YES
	Parkinson	0.132	12	0.200	YES	0.969	12	0.897	YES
p_mean	Healthy	0.162	12	0.200	YES	0.922	12	0.300	YES
	Parkinson	0.258	12	0.027	NO	0.864	12	0.055	YES
p_root_mean	Healthy	0.275	12	0.012	NO	0.716	12	0.001	NO
	Parkinson	0.145	12	0.200	YES	0.916	12	0.258	YES
Variance	Healthy	0.198	12	0.200	YES	0.903	12	0.171	YES
	Parkinson	0.182	12	0.200	YES	0.913	12	0.231	YES
Skewness	Healthy	0.175	12	0.200	YES	0.955	12	0.714	YES
	Parkinson	0.191	12	0.200	YES	0.929	12	0.371	YES
Kurtosys	Healthy	0.265	12	0.020	NO	0.778	12	0.005	NO
	Parkinson	0.287	12	0.007	NO	0.803	12	0.010	NO
Smoothness	Healthy	0.275	12	0.013	NO	0.821	12	0.017	NO
	Parkinson	0.190	12	0.200	YES	0.922	12	0.300	YES

	Class	Kolmogorov-Smirnov test	Shapiro-Wilk test	Descriptive statistics			Levene's test	Student's test	Test mediane (prima riga) / Mann-Withney test (seconda riga)
		Normally distributed?	Normally distributed?	Mean	Standard deviation	Median	p-value	p-value	p-value
**Amplitude**	Healthy	NO	NO	1.764	0.375	29.000	0.001	0.000	0.000
	Parkinson	NO	NO	3.417	0.998	25.500		**0.000**	**0.000**
Duration	Healthy	YES	YES	0.656	1.043	1.732	0.474	**0.476**	0.680
	Parkinson	YES	YES	0.365	0.922	2.976		0.476	0.755
v_mean	Healthy	YES	YES	-0.053	1.673	17.000	0.379	**0.702**	1.000
	Parkinson	YES	YES	-0.291	1.300	8.000		0.702	0.755
**v_max**	Healthy	NO	NO	100.462	14.110	36.578	0.087	**0.000**	0.000
	Parkinson	YES	YES	63.836	21.977	20.096		0.000	**0.000**
**a_max**	Healthy	YES	YES	503.223	77.236	99.334	0.072	**0.001**	0.039
	Parkinson	YES	YES	327.806	135.469	55.154		0.001	**0.006**
jerk_max	Healthy	NO	NO	-1.384	0.836	464.258	0.568	**0.791**	0.684
	Parkinson	NO	NO	-1.505	1.335	241.949		0.792	0.478
Simmetry	Healthy	YES	YES	44.704	0.659	-1.149	0.073	**0.881**	1.000
	Parkinson	YES	YES	44.755	0.966	-1.146		0.881	0.843
p_mean	Healthy	YES	YES	4226.251	94.567	-12.437	0.185	**0.983**	1.000
	Parkinson	NO	YES	4224.869	194.277	-12.319		0.983	0.713
**p_root_mean**	Healthy	NO	NO	114.649	12.894	154.675	0.068	**0.010**	0.039
	Parkinson	YES	YES	96.421	18.370	151.775		0.011	**0.008**
Variance	Healthy	YES	YES	-0.082	0.104	108.718	0.110	**0.190**	0.684
	Parkinson	YES	YES	-0.157	0.159	71.620		0.192	0.410
**Skewness**	Healthy	YES	YES	1.855	0.163	0.412	0.001	0.000	0.003
	Parkinson	YES	YES	2.447	0.423	0.227		**0.000**	**0.000**
**Kurtosys**	Healthy	NO	NO	34.848	0.720	1.761	0.050	0.000	0.000
	Parkinson	NO	NO	37.228	1.025	2.081		**0.000**	**0.000**
**Smoothness**	Healthy	NO	NO	34.226	0.543	35.083	0.001	0.000	0.000
	Parkinson	YES	YES	37.311	1.741	36.900		**0.000**	**0.000**
